# Nutritional Performance of Five Citrus Rootstocks under Different Fe Levels

**DOI:** 10.3390/plants12183252

**Published:** 2023-09-13

**Authors:** Maribela Pestana, Pedro García-Caparrós, Teresa Saavedra, Florinda Gama, Javier Abadía, Amarilis de Varennes, Pedro José Correia

**Affiliations:** 1 MED—Mediterranean Institute for Agriculture, Environment and Development, CHANGE–Global Change and Sustainability Institute, Faculty of Science and Technology, University of Algarve, Campus of Gambelas, Building 8, 8005-139 Faro, Portugal; tmsaavedra@ualg.pt (T.S.); pcorreia@ualg.pt (P.J.C.); 2 Department of Agronomy, Higher Engineering School, University of Almeria, Agrifood Campus of International Excellence CeiA3, Ctra. Sacramentos/n, La Cañada de San Urbano, 04120 Almería, Spain; 3 GreenCoLab—Associação Oceano Verde, University of Algarve, Campus de Gambelas, 8005-139 Faro, Portugal; 4 Plant Biology Department, Estación Experimental de Aula Dei, CSIC, Av. Montañana 1005, 50059 Zaragoza, Spain; 5 Instituto Superior de Agronomia, University of Lisbon, Tapada da Ajuda, 1349-017 Lisbon, Portugal

**Keywords:** citrus, Fe chlorosis, ferric chelate reductase, mineral composition

## Abstract

Iron is an essential micronutrient for citrus, playing an important role in photosynthesis and yield. The aim of this paper was to evaluate the tolerance to Fe deficiency of five citrus rootstocks: sour orange (S), Carrizo citrange (C), *Citrus macrophylla* (M), Troyer citrange (T), and Volkamer lemon (V). Plants were grown for 5 weeks in nutrient solution that contained the following Fe concentrations (in µM): 0, 5, 10, 15, and 20. At the end of the experiment, biomass (dry weight—DW), leaf area, total leaf chlorophyll (CHL), and the activity of root chelate reductase (FCR) were recorded. Additionally, the mineral composition of roots (R) and shoots (S) was evaluated. Principal component analysis was used to study the relationships between all parameters and, subsequently, the relations between rootstocks. In the first component, N-S, P-S, Ca-S, Cu-S, Zn-S, Mn-S, Zn-R, and Mn-R concentrations were related to leaf CHL and FCR. Increases in leaf CHL, Mg-R, and DW (shoots and roots) were inversely related to Cu-R, which was shown in the second component. The values obtained were consistent for V10, C15, and C20, but in contrast for S0 and S5. In conclusion, micronutrient homeostasis in roots and shoots of all rootstocks were affected by Fe stress conditions. The Fe/Cu ratio was significantly related to CHL, which may be used to assist rootstock performance.

## 1. Introduction

Plants have different adaptation responses to nutrient depletion, such as improvement in root nutrient uptake, reallocation of nutrients to priority organs, and modulation of cellular physiology [1]. Iron (Fe) deficiency is the most common micronutrient deficiency in calcareous soils. In the Mediterranean basin, it has been estimated that 20–50% of fruit orchards have Fe chlorosis [2], which causes reduced yield and quality [3]. Symptoms always start on young leaves with an interveinal chlorosis, where the veins remain greener than the rest of the leaf [4].

When faced with Fe deficiency, plants can be grouped into two strategies, according to their induced Fe uptake mechanisms [5]: Strategy I, considered the reduction-based strategy, typical of non-Poaceae species, and Strategy II, or complexing strategy, characterized by the formation of soluble Fe(III) complexes which are then taken up by roots. The latter is found exclusively in monocotyledonous plants of the Poaceae family. Furthermore, rice (*Oryza sativa* L.) plants employ a combined strategy, composed of all features of Strategy II and some features of Strategy I [6]. Recently, a new approach indicated that the boundaries between these two strategies are not so well defined, since dicotyledons can also exudate complex Fe compounds [7] and grasses can have the ability to reduce Fe [8].

Citrus, like the other dicots, are included in the Strategy I group, which comprised several steps to increase Fe uptake into roots, such as proton extrusion, secretion of chelators, enhancement of Fe(III) reduction, and increased activity of Fe transporters in the root plasmalemma [4]. These steps occur through an increase in rhizosphere acidification (H^+^-ATPase) and in the activity of ferric chelate reductases (FCRs), and, consequently, in the uptake of Fe^2+^ through the root cell membranes, mediated by IRT1, a specific regulated Fe transporter [9,10]. After the uptake into rhizodermal cells, Fe moves across the cortical cells towards the xylem vessels in a chelated form [11]. Once in the leaves, Fe is essential in several metabolic processes such as photosynthesis, respiration, and nitrate reduction [12].

Iron homeostasis is controlled not only by root Fe availability but also by leaves that, under chlorosis, are able to send signals to the roots to induce the reactions described above [10]. Rootstocks/scion combinations determine tree size, yield, and fruit quality parameters and may be used to enhance tolerance to specific abiotic stress. Citrus resistance to Fe deficiency is primarily determined by the rootstock and its ability to trigger physiological, biochemical, and morphological mechanisms at root level (for a review see [4,10]).

Rootstocks that combine tolerance to Fe deficiency with resistance to biotic stress (such as Tristeza virus or *Phytophthora* spp.) are still scarce but there is a large pool of work on the response of citrus rootstocks to several abiotic and nutritional stresses [13,14,15,16]. In this regard, Martínez-Cuenca et al. [13] compared Carrizo citrange (Fe chlorosis sensitive) and Cleopatra mandarin (Fe tolerant) and concluded that the later accumulated more Fe in the root apoplast.

In a recent review by Alfaro et al. [17], it was concluded that citrus rootstocks influence the quality of citrus fruits; however, few studies have focused on the assimilation of nutrients by rootstocks or on the corresponding translocation of nutrients through the rootstock/cultivar union. More recently, the particular importance of Fe in citrus was demonstrated, since the application of Fe(II) could restore the growth of trees affected by citrus greening or Huanglongbing (HLB), one of the most serious citrus diseases in the world [18].

In our previous work, five citrus rootstocks were classified according to their resistance to Fe deficiency [19]. This approach was based on the number of days that each plant material was able to grow under low Fe concentrations in the nutrient solution before the leaf chlorophyll (CHL) concentration decreased to 50%. Under the experimental conditions of the study, sour orange and Volkamer lemon were able to cope with low Fe for a longer period and were classified as tolerant to Fe deficiency.

Iron deficiency affects multiple metabolic pathways, including nutrient uptake and transport to the leaves, which lead to unbalanced mineral composition in different organs. Several studies (for example [4,20,21,22]) were conducted to access the effect of Fe chlorosis on the nutritional patterns in citrus but, in some cases, the results were inconsistent, since nutritional requirements are highly variable.

The aim of this work was to compare Fe use in five citrus rootstocks (Troyer citrange (*Citrus sinensis* (L.) Osb. × *Poncirus trifoliata* (L.) Raf.), Carrizo citrange, Volkamer lemon (*C. volkameriana* Ten. & Pasq.), alemow (*C. macrophylla* Wester), and sour orange (*C. aurantium*) when grown under different Fe levels, and to establish the impact of Fe chlorosis on nutritional composition of shoots (S) and roots (R). It was expected that this work would reveal some trends in the ability of citrus rootstocks to cope with Fe deficiency.

## 2. Results

At the beginning of the experiment, values of leaf CHL (Table S1), ranged between 349.9 ± 78.0 µmol m^−2^ (sour orange) and 460.2 ± 93.1 µmol m^−2^ (Troyer citrange) and shoot dry weight (DW) presented values between 0.29 ± 0.02 g (Troyer citrange) and 0.66 ± 0.07 g (sour orange).

The root DW varied between 0.09 ± 0.01 g (*C. macrophylla*) and 0.22 ± 0.01 g (Carrizo citrange). The lowest root-to-shoot ratio (DW) value was observed in *C. macrophylla* (0.30 ± 0.07 g) and the highest in Troyer citrange (0.62 ± 0.13 g). The total plant DW (shoot plus roots) ranged between 0.39 ± 0.01 g (*C. macrophylla*) and 0.87 ± 0.04 g (sour orange).

### 2.1. Chlorophyll Concentration, Biomass and Root Ferric Chelate Reductase Activity

At the last day of the experimental period, physiological responses to Fe availability in the nutrient solution varied between rootstocks (Table 1; Figure S1).

Iron concentration in nutrient solution did not affect total plant DW in either the sour orange or the Troyer rootstocks. Nevertheless, Volkamer lemon plants increased DW under higher Fe concentrations in the nutrient solution, while the other two citrus rootstocks did not show a clear trend. The root/shoot ratio (in DW) was only affected in sour orange and *C. macrophylla* but without obvious trends.

At the end of the experiment, all the rootstocks grown with 20 µM of Fe, except Carrizo citrange, showed greater leaf area (cm^2^) and leaf CHL concentrations (positive values in % of variation between initial and final values). The DW of plants of all treatments and rootstocks was also incremented considering the initial DW values, particularly in plants grown with higher Fe. Regardless of the rootstock, the symptoms of Fe chlorosis appeared whenever the decrease in foliar CHL concentration was greater than 25% of the initial values. Leaf CHL values ranged from a minimum of 39.9 µmol m^−2^ (−91.3%; Troyer citrange) to a maximum of 212.9 µmol m^−2^ (−45.0%; Volkamer lemon). Leaf area of all rootstocks also increased as a response to Fe treatments. This was less clear in sour orange, as significant differences were only observed between treatments with 0 and 20 µM of Fe.

Iron leaf chlorosis was observed at concentrations of 0, 5, 10, and 15 µM of Fe for the rootstocks Carrizo citrange, *C. macrophylla*, Troyer citrange, and sour orange, while in Volkamer lemon it was only observed at the two lowest concentrations (0 and 5 µM of Fe).

The root FCR activity of Carrizo and Troyer rootstocks reached maximum values at the concentration of 15 µM of Fe. In *C. macrophylla*, FCR activity was induced in plants grown in the total absence of Fe, while in the remaining rootstock there was no consistent pattern.

### 2.2. Mineral Composition

The concentrations of macronutrients and micronutrients in the shoots are shown in Table 2. In sour orange, P and Fe concentrations in shoots showed a clear increase in treatments with higher Fe levels. The concentrations of N, Ca, Mg, and Cu did not show significant differences between treatments.

In Carrizo citrange, Ca, Mg, K, and Fe concentrations were similar between treatments. Phosphorus was higher in treatments with 10, 15, and 20 µM of Fe. Copper and Zn did not show a clear pattern, and Mn was significantly higher at 15 µM of Fe.

In shoots of *C. macrophylla*, the concentrations of Mg, Fe, and Cu were similar, while that of Mn was significantly higher in the absence of Fe in the solution. Calcium, K, and Zn did not show a clear trend, but P was higher in the treatment of 15 µM of Fe.

Troyer citrange did not show significant differences in the concentrations of N, Ca, Mg, and Zn in the shoots. The highest concentrations of Fe and K in the shoots were registered in the treatments with 20 and 10 µM of Fe, respectively.

In Volkamer lemon, K and Cu concentrations in the shoots were similar between treatments. Phosphorus was higher in the treatments with 10, 15, and 20 µM of Fe, and Zn in 10 µM of Fe. The Fe concentration in shoots was higher in treatments with 15 and 20 µM of Fe.

In the case of the mineral composition of the root (Table 3), there were different responses in the studied citrus rootstocks. For example, in sour orange, N and Zn concentrations in the roots were similar. Regarding the other nutrients evaluated, the values of P, Ca, and Mg were higher at 15 µM of Fe, while K, Fe, and Mn were higher at 20 µM of Fe. On the other hand, Cu increased when no Fe was present in the nutrient solution.

Carrizo citrange did not show a clear trend regarding N, Mg, or Fe. However, P, K, and Zn were higher in the treatment with 10 µM Fe, and Ca in the 15 µM Fe treatment. Manganese concentration in the roots was higher in the treatment with 20 µM of Fe, but the concentration of Cu in the roots was higher in the treatments with 0, 5, and 10 µM of Fe.

In *C. macrophylla*, the root concentrations of N, Mg, and K remained the same in all treatments, while those of Cu, Zn, and Mn were higher in the treatment without Fe. Calcium did not present a clear response, and P was higher at 20 µM of Fe in the nutrient solution. Root Fe concentration was significantly higher at 5 and 10 µM of Fe.

Troyer citrange did not show significant differences in the concentration of N in the root. No clear response was observed for P, Ca, Mg, Fe, and Mn, but Zn and Cu concentrations were higher in the absence of Fe. Root K concentration increased in treatments with higher levels of Fe (15 and 20 µM of Fe).

In Volkamer lemon, the Fe concentration in the roots remained constant regardless of the treatment. Root concentrations of N, K, and Mn were higher in the treatments with more Fe, while Cu values was significantly higher in roots grown without Fe. No evident response was detected for P and Ca.

The principal component analysis (PCA) analysis selected six principal components with eigenvalues greater than 1 among the 23 parameters tested, including shoot and root nutrient composition, DW, leaf CHL, leaf area, and root FCR. The cumulative percentage variance of the six principal axes was 85.2% of the total variance. 

**Figure 1:**
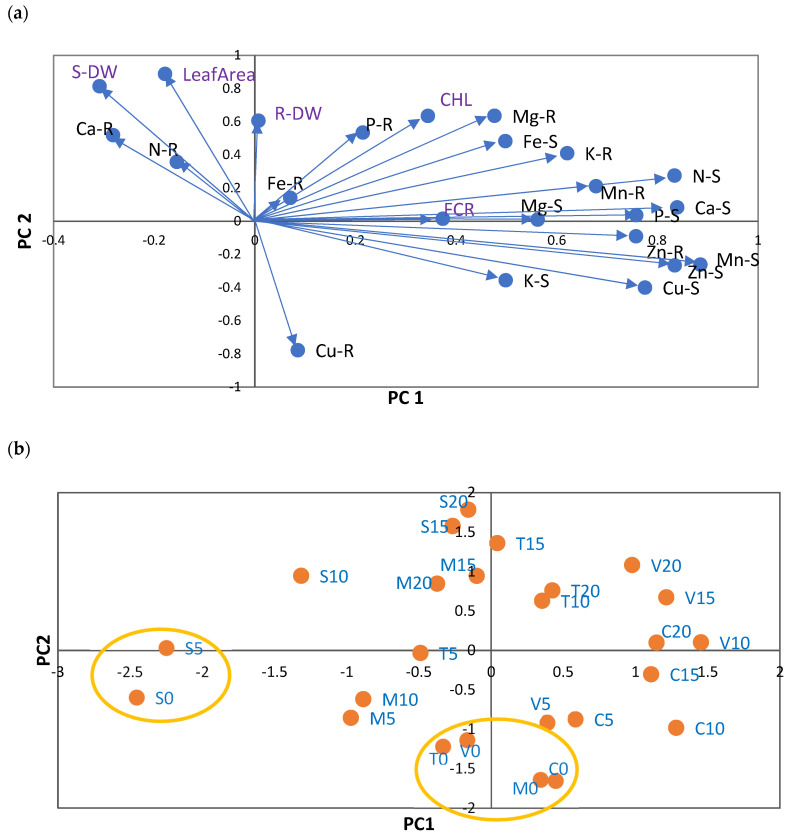
(**a**) Principal component analysis of nutrient concentrations (in mg g^−1^ DW: N, P, K, Ca, and Mg; in µg g^−1^: DW: Fe, Cu, Zn, and Mn) in shoots (S) and roots (R) of five citrus rootstocks. PC1—first principal component; PC2—second principal component. Each vector represents the loadings of variables (nutrients and Fe chlorosis parameters: CHL, leaf area, S-DW, R-DW, and FCR) in each principal component. Loadings represent the relative contribution of each nutrient to that component. Concentration of nutrients in shoots (S) and roots (R): N (N-S and N-R), P (P-S and P-R), K (K-S and K-R), Ca (Ca-S and Ca-R), Mg (Mg-S and Mg-R), Cu (Cu-S and Cu-R), Fe (Fe-S and Fe-R), Mn (Mn-S and Mn-R), and Zn (Zn-S and Zn-R). (**b**) Projection of 25 scores resulting from 5 rootstocks (S —sour orange, V—Volkamer lemon, T—Toyer citrange, C—Carrizo citrange and M—*C. macrophylla*) × 5 Fe levels (0, 5, 10, 15, and 20 µM of Fe) onto the plane defined by the principal components. The labels of treatments result from the combination of the letter associated with each rootstock and the concentration of Fe in nutrient solution.

The variations in nutrient concentrations in shoots and roots for the five citrus rootstocks produced a dominant first principal component (PC1), which explained 31% of the total variance (Figure 1a; Table S2).

The second component (PC2) explained 21% of the variance and PC3 (third component) explained 13%, with further components explaining less than 8%. This analysis indicated that data could be summarized in two dimensions. Increases in N-S, P-S, Ca-S, Cu-S, Zn-S, Mn-S, Zn-R, and Mn-R concentrations were coordinated with CHL and FCR, and placed in contrast to S-DW along PC1. However, the contribution of S-DW was small. PC2 reflected the coordinated increases in leaf CHL, Mg-R, and DW (shoots and roots) in contrast to Cu-R.

Along PC1 and PC2, the scores for each treatment (rootstock × Fe level = 25) were also analyzed (Figure 1b). The main patterns identified along the two principal components (PC1 and PC2) were mainly related to differences between Fe treatments. Along PC1, the values obtained were consistent for V10, C15, and C20, but opposite to those obtained for S0 and S5. Consequently, the Fe0 level had the highest concentrations of N-S, P-S, Ca-S, Cu-S, Zn-S, Mn-S, Zn-R, and Mn-R, and the lowest values of S-DW. Regarding PC2, it was evident that S20, S15, T15, M15, and M20 presented the smallest values of Cu-R.

As expected, several significant relationships were observed between the studied parameters, as shown in Pearson’s correlations (Table S3). CHL leaf concentration was positively related to Fe-S (0.56) and K-S (0.51), but inversely related to Cu-R (−0.53). Root FCR was directly related to K-S (0.63) and Mn-S (0.41).

**Figure 2:**
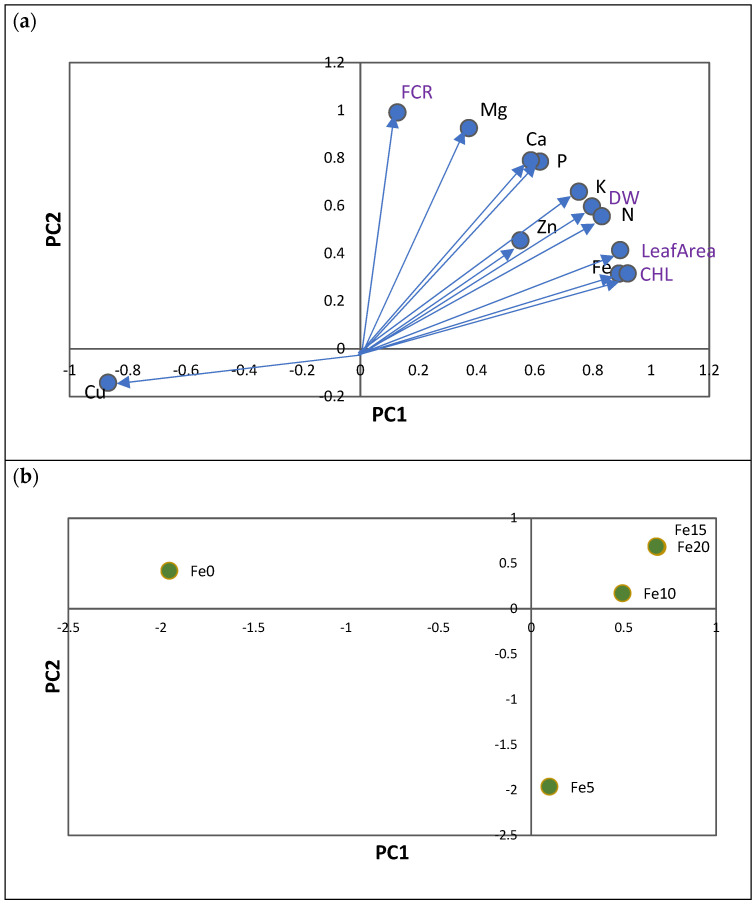
(**a**) Principal component analysis of nutrient contents (in mg: N, P, K, Ca, and Mg; and in µg: Fe, Cu, Zn, and Mn) of five citrus rootstocks considering the total in plants (roots plus shoots). PC1—first principal component; PC2—second principal component. Each vector represents the loadings of variables (nutrients and Fe chlorosis parameters: CHL, leaf area, S-DW, R-DW, and FCR) in each principal component. Loadings represent the relative contribution of each nutrient to that component. (**b**) Projection of 5 Fe levels (0, 5, 10, 15, and 20 µM of Fe) considering all rootstocks.

To assess the influence of five Fe levels on leaf chlorosis parameters (CHL, FCR, total DW, and leaf area) and nutrient contents (in mg: N, P, K, Ca, and Mg; in µg: Fe, Zn, Cu, and Mn; Table S4) of whole plants, a PCA was performed that considered all rootstocks as one (Figure 2). PCA extracted two axes with eigenvalues >1, representing about 92% of the overall variability in the data. After rotation of the PC axis using the varimax approach (normalized), two axes showed patterns associated with Fe chlorosis variables (Figure 2a; Table S5).

The first axis (PC1), which reflects leaf chlorosis, represented the variation in leaf CHL concentration, leaf area, and total DW of plants. CHL leaf concentration was positively associated with increases in total Fe content in plants and with increases in N, P, K, Mg, and Zn contents, and with decreases in total Cu contents (Figure 2a). The root FCR variable was represented in a separate axis (PC2) and was shown to be totally unrelated to the Fe chlorosis variables. Increases in FCR were associated with increases in Ca and Mn contents in plants (Figure 2a).

The main patterns identified along the two principal components (PC1 and PC2) were mainly related to the differences between treatments with and without 5 µM of Fe (Fe0 and Fe5; Figure 2b). Consequently, the plants in the treatments without Fe (Fe0) showed the lowest values of leaf CHL, leaf area, and total DW, which were associated with the lowest content of N, P, Mg, K, Fe, and Zn and the highest content of Cu. In PC2, it is evident that treatments with 5 µM Fe (Fe5) showed the lowest values of FCR, and Ca and Mn contents.

**Figure 3:**
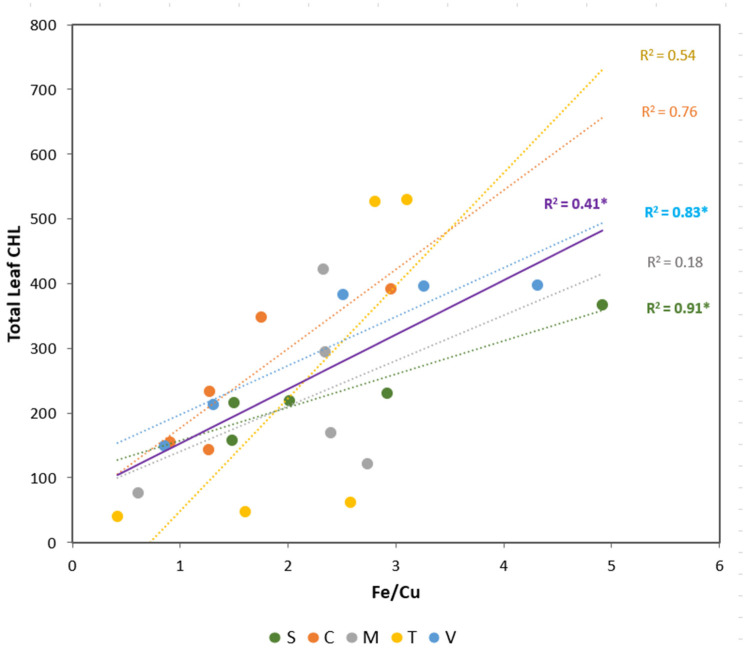
Linear regression relationships between total leaf CHL (µmol m^−2^) and total Fe/Cu ratio for each rootstock (circles) and considering all treatments together (purple line). Treatments are 5 rootstocks (S—sour orange, V—Volkamer lemon, T—Toyer citrange, C—Carrizo citrange and M—*C. macrophylla*) × 5 Fe levels (0, 5, 10, 15, and 20 µM of Fe). The coefficient of determination (R^2^) is presented for each model and significant models (*p* < 0.05) are indicated by one asterisk (*).

As Cu showed a contrasting response between macro and micronutrients (Figure 1a and Figure 2a), the Fe/Cu ratio was studied in detail. A positive linear relationship was obtained between the Fe/Cu ratio and foliar CHL concentrations (Figure 3).

This global analysis highlights the relationship between the Fe/Cu ratio and leaf CHL concentration (R^2^ = 0.41). However, when we analyzed this relationship in each citrus rootstock, only Volkamer and sour orange plants showed a significant relationship between these parameters. These rootstocks exhibited the highest Fe/Cu ratio (>4) at 20 µM of Fe. On the other hand, the lowest values were registered in the absence of Fe for Troyer and *C. macrophylla* plants (<1).

## 3. Discussion

In citrus production, selecting the adequate rootstock is a key element not only to face adverse environmental conditions, including biotic and abiotic stress, but also because it influences the final quality of the fruits (for a review, see Alfaro et al. [17]). Although there are previous works on the physiological responses of Fe-chlorotic citrus plants [19,21], it is of great importance to establish the nutritional profile of macro- and micronutrients of citrus rootstocks grown under different Fe intakes to improve their nutritional performance and select the best scion/rootstock combinations.

In previous results [19], we identified two strategies associated with the growth and Fe-use efficiency of these species: an Fe-spending strategy versus an Fe-conservative strategy.

Volkamer, a non-trifoliate species, is a fast-growing rootstock that is able to cope with Fe deficiency by promoting increased Fe reductase activity in the roots [19]. The FCR results presented here are inconclusive, but there was a nutritional adaptation to Fe stress conditions. In fact, in this rootstock, the highest proportions of nutrients were allocated to the shoot compared to the roots, probably due to efficient upward transport, which may have promoted photosynthetic efficiency and/or a better performance of antioxidative systems, as suggested by Oustric et al. [23]. Interestingly, the absence of a clear response of root nutrients profiles in Volkamer may indicate that Fe depletion did not have an impact on nutrient accumulation in roots.

Sour orange also presents tolerance to Fe depletion [19]. Only Fe and P were notably enhanced in shoots due to the increment in Fe in the solution (15 and 20 µM Fe treatments), suggesting that small amounts of Fe were not enough to trigger some nutritional imbalance in the shoot. However, Fe stress signaling occurred at the root level, as Cu was increased in response to the total absence of Fe, which led to a lower Fe/Cu ratio. This close functional role between these two metals (Fe and Cu) has already been reported in several species [24,25,26].

The rootstock most sensible to Fe deficiency is Troyer citrange, a fact supported by the very low values of leaf chlorophyll registered at the end of the experiment. Regarding this rootstock, most of the nutrients present in the shoot, with the clear exception of N, were partially affected by Fe deficiency, but the pattern was quite inconclusive (for example, the concentrations of all micronutrients were higher in the treatments 10 and 15 µM of Fe). However, in the roots, it is possible to observe that, in addition to Cu, Zn also accumulated in the treatments without Fe. This specific increase in Zn in the root was also observed in the susceptible *Poncirus trifoliata* [20].

*Citrus macrophylla* and Carrizo citrange can be considered rootstocks with an intermediate resistance to Fe depletion. In *C. macrophylla*, the main outcome under Fe depletion was the increase in Zn and Mn in the shoots, and in Cu, Zn, and Mn in the roots. It is possible that these nutrients were taken up instead of Fe, a strategy already mentioned as a response to Fe deficiency. However, the increment in Mn in the roots is a trait that distinguished this rootstock from the others. Although Mn activates many enzymes, only a small number contain Mn [27]. One of these is superoxide dismutase (MnSOD), which plays a role in cell protection from oxygen radicals. It can be assumed that the lack of Fe induced an oxidative stress and, consequently, there was a greater demand for Mn for the activity of the SOD enzyme.

Carrizo citrange is also moderately resistant to Fe chlorosis and it was found that, as Fe availability increased, the concentration of several macro- and micronutrients also increased both in shoots and roots, thus supporting the negative and generalized impact of low Fe values in its mineral composition. However, like in *C. macrophylla*, higher concentrations of Cu were observed in the treatment without Fe. FCR activity was low in plants without Fe in the solution, but similar to that of the treatment with 20 µM of Fe, which is not in accordance with the findings of Martinez-Cuenca et al. [13].

In citrus, the processes involved in tolerance to Fe chlorosis depend on the genotype [23]. Fu et al. [28] found that Fe tolerance was associated with, among other factors, increased Fe uptake in roots and subsequent translocation to shoots. In our experiment, the Volkamer rootstock was able to allocate a higher percentage of Fe to shoots, suggesting an efficient use of Fe. In fact, a high Fe content in the roots was one of the traits identified by Forner-Alcaide [29] as being associated with greater tolerance to Fe deficiency in citrus rootstocks.

Considering all rootstocks, the PCA outcome (PC1) revealed a coordinated pattern of most of the nutrients in shoots and leaf CHL, which supports the metabolic link between photosynthesis and nutrient uptake and transport. However, it is clear that the concentration of Cu in roots behaves differently (as shown by the contrasting pattern with total DW and leaf CHL), which highlights the importance of Cu in Fe stress response.

The PCA analysis of the rootstocks confirmed that the nutritional balance of sour orange when grown with low Fe availability (0 and 5 µM of Fe) contrasted with that of the other rootstocks that grew at the highest Fe concentrations (10, 15, and 20 µM of Fe).

It seems that in the absence of Fe in the nutrient solution, although Cu can be taken up in place of Fe, it cannot fulfil the functions of Fe, compromising the entire performance of the plants.

Metal homeostasis plays an important role in plant growth, with a complex network of interactions. Iron and Cu metabolic pathways are closely linked as they share the same metal transporters (such as IRT1) and metal-binding proteins. For example, nicotianamine is a non-proteinogenic amino acid able to bind to Fe, but which also has high affinity to other metals such as Cu [30]. Interaction between Fe and Cu is well documented. For example, Waters and Armbrust [31] found that, in Arabidopsis, leaf Cu concentration increased under low Fe supply, and that high Cu reduced FCR activity. Thus, several reports suggest that Fe-deficient plants assimilate extra Cu to supply CuSOD proteins and ensure protective mechanisms against oxidative damage [32].

Rootstocks’ responses demonstrated how Fe deficiency induces imbalances in citrus trees, thus highlighting the importance of establishing the best nutrient management for sustainable fruit production. In addition, it will be possible to control Fe applications using rootstocks with greater Fe use efficiency and adapting fertilization programs for more sustainable production.

We can conclude that the reduced uptake of mineral elements under Fe depletion is not only due to the lack of Fe but also to the balance between nutrients. Therefore, total Cu seems to be a key nutrient to distinguish Fe tolerance, considering the set of all rootstocks under study. Furthermore, the Fe/Cu ratio is useful to elucidate the difference in the use of these micronutrients among rootstocks under different Fe availability, as well as to evaluate the impact of this nutritional balance on foliar CHL synthesis.

It remains necessary to investigate the role of rootstocks on the impact caused by Fe deficiency on fruit production and quality, but the outcome of this experiment may also allow the refinement and optimization of nutritional management under Fe stress conditions. Likewise, continuous screening of genotypic varieties with high resistance to iron deficiency is essential to overcome plant tolerance to Fe deficiency.

## 4. Materials and Methods

### 4.1. Plant Material and Growth Conditions

The experiment was conducted with five citrus rootstocks grown under greenhouse conditions. The five citrus rootstocks studied were Troyer citrange (*Citrus sinensis* (L.) Osb. × *Poncirus trifoliata* (L.) Raf.), Carrizo citrange, Volkamer lemon (*Citrus volkameriana* Ten. & Pasq.), alemow (*C. macrophylla* Wester), and sour orange (*C. aurantium* L.). Seeds of all rootstocks were obtained from Willits & Newcomb (Arvin, CA, USA), and were disinfected by immersion in a 15% of sodium hypochlorite solution for 15 min and then washed three times with distilled water.

Seeds were germinated in the dark at 22 °C in plastic trays with sterilized moist vermiculite. After germination, seedlings were grown in moist vermiculite for four weeks in a controlled environment with day/night temperatures of 21/22 °C, a relative humidity of 80%, and a 12 h photoperiod. During this period, the minimum photon flux density at plant level was 113 μmol quanta of photosynthetic active radiation (PAR) m^−2^ s^−1^, provided by a combination of fluorescent and incandescent lamps.

After this period, groups of uniform seedlings (14 ± 2 cm length and five to nine fully expanded leaves) were selected for each rootstock and transferred to 20 L polyethylene vessels (six plants per vessel) filled with full-strength Hoagland solution containing (in mM): 5.0 Ca(NO_3_)_2_, 1.4 KNO_3_, 0.6 K_2_SO_4_, 1.0 MgSO_4_, 0.9 NaCl, 0.6 (NH_4_)_2_HPO_4_, 3.0 (NH_4_)_2_SO_4_, 0.2 MgCl_2_ and (in µM): 41.8 H_3_BO_3_, 3.8 ZnSO_4_, 3.9 CuSO_4_, 6.9 MnSO_4_, and 1.0 (NH_4_)_6_Mo_7_O_24_ following the recommendations established by Carpena [33]. Iron was added to the solution as Fe(III)-EDHHA at five different concentrations (µM Fe): 0 (Fe0), 5 (Fe5), 10 (Fe10), 15 (Fe15), and 20 (Fe20). Treatments were imposed for five weeks. The initial pH of nutrient solutions was adjusted to 6.0 ± 0.1 using NaOH 0.1 M (the electrical conductivity (EC) was 2.1 ± 0.1 dS m^−1^). The pH and EC of the solutions were monitored every two days. The nutrient solution was frequently aerated, using alternated cycles of 15 min with and without aeration programmed with a timer. The experiment was performed between June and July in a glasshouse under natural photoperiod conditions. The air temperature was ≤25 °C and the average relative humidity was 65%. The experiment was arranged in a complete randomized design, with 25 combinations of Fe concentrations and rootstocks, and six replications (plant) per combination.

Measurements of different parameters were carried out immediately at the beginning and at the end of the experiment, in at least five plants per treatment (each Fe level and each rootstock).

### 4.2. Leaf Chlorophyll

Leaf chlorophyll (CHL) concentrations were estimated using a portable SPAD-502 apparatus (Minolta Corp., Osaka, Japan). The SPAD-502 readings were taken in the youngest fully expanded leaves, using the average of at least three measurements per leaf. Leaf SPAD readings were converted to total CHL concentrations (in µmol m^−2^) using the same calibration equations, i.e., quadratic regression models, obtained in a previous study with the same citrus rootstocks [19]. Leaf CHL values were extracted using 100% acetone in the presence of Na ascorbate according to the method described by Abadía and Abadía [34]. The percentage of variation between initial leaf CHL concentration and final values was also calculated.

### 4.3. Determination of the Root Fe Chelate Reducing Capacity

The activity of the root ferric chelate reductase (FCR; EC1.16.1.17) was measured at the end of the experiment (after 34 days) in all plants, using bathophenanthroline disulfonate (BPDS), which forms a red Fe_3_BPDS_2_ complex with Fe(II), as in Bienfait et al. [35]. In each plant, a single root tip, approximately 2 cm in length (14.39 ± 0.92 mg of dry weight—DW), was excised with a razor blade and incubated in an Eppendorf tube, in darkness for one hour, with 900 μL of micronutrient-free half-strength Hoagland’s nutrient solution, containing 300 µM BPDS, 500 μM Fe(III)-EDTA (EDTA-ethylenediamine-tetraacetic acid), and 5 mM MES (2-(N-morpholino)ethanesulfonic acid), pH 6.0. Then, the FCR activity was measured at 535 nm, using a spectrophotometer (CADAS 100 UV-VIS Photometer; Dr. Lange, Düsseldorf, Germany), with a molar extinction coefficient of 22.14 mM^−1^ cm^−1^. Each root tip was then blotted on paper and the fresh weight (FW) was determined. Values shown for FCR activity were the mean of at least five root tips (plants) in each treatment, and values were calculated on a FW basis. Blank controls without root tips were also used to correct for any non-specific photoreduction.

### 4.4. Biomass Determination, Leaf Area, and Mineral Composition Analysis

At the beginning of the experiment, plants of each rootstock and Fe level were collected and separated into shoots (leaves and stems) and roots. The leaf area was measured in at least 5 plants per treatment and per rootstock, considering the set of leaves of each plant. For this measurement, a leaf area meter was used (ADC, AM 200). Each sample consisted of two plants from each treatment and three replicates were analyzed.

The plant material was weighed to determine the fresh weight (FW) and subsequently washed with tap water, followed by deionized water with a non-ionic detergent and then with 0.01 M HCl. To finish, three rinses were made with distilled water. The plant material was dried at 70 °C for at least 48 h until a constant weight was reached to determine the respective dry weight (DW). Dried samples were ground to pass through a 1 mm stainless sieve and stored. Nitrogen and P were determined by the Kjeldahl method and colorimetrically by the molybdovanadate method, respectively. The dried material was ashed at 450 °C, followed by digestion with 1 M HCl. Potassium was determined by emission spectrophotometry, and Ca, Mg, Mn, Zn, Cu, and Fe were determined by atomic absorption spectrophotometry (SolaarM Series, Pye Unicam, Cambridge, UK) following standard laboratory procedures [36]. Macronutrient concentrations were expressed as mg g^−1^ DW and micronutrients as µg g^−1^ DW. Nutrient contents were calculated by multiplying the DW of each plant organ by the nutrient concentration in that organ. The total nutrient contents resulted from the sum of the values obtained in shoots (leaves and stems) plus roots. The nutrient ratio was calculated using the total content of the different nutrients assessed per citrus rootstock.

### 4.5. Statistical Analysis

The experiment had a completely randomized design. Analysis of variance (ANOVA; F test) was conducted for all studied variables and conditions, and the means were compared using the Duncan Multiple Range Test at *p* ≤ 0.05.

The assessment of the main nutritional patterns as a response to all treatments (5 Fe levels × 5 rootstocks) was conducted using principal component analysis (PCA). Using PCA, it is possible to reveal associations in the data that cannot be found by analyzing each variable separately. Each extracted component or factor accounts for part of the variation in all data sets and is associated with an eigenvalue. The eigenvalue associated with each eigenvector is a measure of the variance within variables of the corresponding principal component. The eigenvectors can be used to calculate new values, called scores, for each observation on each principal component. The scores can be positioned on a plot to identify the cases that contributed more to the formation of the component. For the interpretation of data, only the components with eigenvalues greater than one were kept (Kaiser’s criterion). To obtain a better representation of gradients in shoot and root nutrients associated with chlorosis variables (CHL, FCR, shoot DW, and root DW), a varimax (normalized) rotation was applied to the PCA results. This approach simplifies interpretation of the patterns associated with any given parameter of interest because the rotation maximizes the loading of each parameter in a single factorial axis.

In addition, PCA was also carried out to determine the relationships between the mineral composition according to the Fe level regardless of the rootstock. For this PCA, new averages per rootstock resulted from the grouping of nutrient content by Fe level.

Linear correlations between studied parameters were determined and the Pearson correlation coefficients presented. For each rootstock, linear regression analysis between leaf CHL on the last day and the plant Fe/Cu ratio was carried out. The global model for all rootstocks was also presented.

Statistical analyses were performed using the SPSS^®^ software (IBM SPSS Statistics for Windows, Version 29.0. Armonk, NY, USA, IBM Corp).

## Figures and Tables

**Table 1:** Total plant dry weight (DW in g and % of variation between initial and final values), root-to-shoot ratio (in DW basis), total leaf chlorophyll concentration (CHL in µmol m^−2^ and % of variation between initial and final values), leaf area (cm^2^), and root ferric chelate reductase activity (FCR in nmol Fe(II) min^−1^ g^−1^ fresh weight—FW) at the end of the experiment of five citrus rootstocks grown in different Fe concentrations: 0, 5, 10, 15, and 20 µM.

Treatments	Plant DW		Root/Shoot	Leaf CHL		Leaf Area	Root FCR
[µM Fe]	(g)	% Variation		* µmol m^−2^	% Variation	(cm^2^)	(nmol Fe(II) min^−1^ g^−1^ FW)
Sour orange							
0	1.58 a	82%	0.28 c	157.9 b	−54.9%	104.0 b	0.66 ab
5	1.83 a	110%	0.36 ab	214.9 b	−38.6%	119.0 ab	0.40 b
10	1.98 a	128%	0.31 bc	218.9 b	−37.4%	135.5 ab	0.78 ab
15	1.93 a	121%	0.45 a	230.7 b	−34.1%	151.5 ab	1.79 ab
20	1.82 a	110%	0.42 ab	367.3 a	4.9%	179.1 a	1.83 a
Carrizo citrange							
0	0.90 b	96%	0.59 a	154.9 c	−66.0%	43.9 c	1.01 b
5	1.22 a	44%	0.60 a	142.8 c	−68.7%	62.6 bc	1.73 b
10	1.06 ab	72%	0.59 a	232.3 b	−49.0%	69.7 bc	1.15 b
15	1.18 a	92%	0.60 a	348.1 a	−23.6%	90.2 b	5.02 a
20	1.28 a	106%	0.76 a	391.4 a	−14.1%	120.7 a	1.56 b
*C. macrophylla*							
0	0.87 c	121%	0.59 b	76.2 d	−81.0%	50.4 b	6.48 a
5	0.77 c	96%	0.76 a	169.3 c	−57.7%	55.7 b	0.62 c
10	0.96 c	145%	0.59 b	120.7 cd	−69.8%	111.4 a	1.10 c
15	1.63 a	318%	0.60 b	294.6 b	−26.3%	120.3 a	4.55 ab
20	1.29 b	231%	0.49 b	421.4 a	5.4%	146.3 a	3.87 b
Troyer citrange							
0	1.48 a	214%	0.61 a	39.9 b	−91.3%	85.0 c	0.72 c
5	1.56 a	232%	0.56 a	47.4 b	−89.7%	101.8 b	2.07 b
10	1.71 a	264%	0.51 a	61.1 b	−86.7%	148.2 ab	1.24 bc
15	2.07 a	339%	0.50 a	529.1 a	14.2%	174.3 a	6.49 a
20	1.92 a	309%	0.45 a	520.3 a	15.0%	127.2 ab	0.90 c
Volkamer lemon							
0	1.07 c	122%	0.44 a	149.2 b	−61.5%	49.3 b	0.86 b
5	1.31 bc	174%	0.43 a	212.9 b	−45.0%	62.1 b	3.25 ab
10	1.39 bc	191%	0.40 a	381.8 a	−1.4%	108.4 ab	4.68 a
15	1.73 a	260%	0.36 a	395.7 a	2.2%	136.7 a	1.38 b
20	1.93 a	302%	0.36 a	397.6 a	2.7%	164.2 a	2.42 ab

For each rootstock, and each parameter, means with the same letter were not significantly different at *p* < 0.05, using the Duncan’s test. * CHL data were obtained by Pestana et al. [19].

**Table 2:** Macronutrient (mg g^−1^ DW) and micronutrient (µg g^−1^ DW) concentrations at the end of the experiment in shoots of five citrus rootstocks grown in five Fe concentrations:0, 5, 10, 15 and 20 µM.

Treatments [µM Fe]	N	P	Ca	Mg	K	Fe *	Cu	Zn	Mn
Sour orange									
0	24.80 a	1.50 b	11.40 a	1.30 a	10.70 c	37.46 b	5.90 a	20.70 b	28.57 ab
5	27.46 a	1.35 b	11.80 a	1.45 a	12.70 abc	32.00 b	9.60 a	21.87 ab	18.12 bc
10	30.16 a	1.55 b	11.70 a	1.40 a	11.50 bc	40.36 b	7.09 a	24.56 a	16.37 c
15	31.50 a	2.07 ab	13.20 a	1.65 a	14.80 a	64.70 a	7.96 a	25.98 a	27.59 abc
20	28.95 a	2.49 a	12.35 a	1.45 a	13.60 ab	80.36 a	6.34 a	22.69 a	35.35 a
Carrizo citrange									
0	36.84 b	3.68 b	18.45 a	2.20 a	16.35 a	69.35 a	16.32 ab	45.52 c	68.08 b
5	34.34 b	4.34 b	16.00 a	2.20 a	14.50 a	45.08 a	13.36 b	77.13 a	85.35 b
10	41.35 ab	6.65 a	14.30 a	2.45 a	15.50 a	57.20 a	27.78 a	57.82 bc	91.59 b
15	45.34 a	5.84 a	21.00 a	2.45 a	18.20 a	57.69 a	17.34 ab	66.69 ab	116.98 a
20	40.46 ab	6.10 a	20.00 a	2.15 a	17.45 a	78.15 a	16.94 ab	51.47 c	88.91 b
*C. macrophylla*									
0	28.90 a	2.16 b	18.90 a	1.25 a	25.65 a	50.49 a	18.61 a	46.27 a	115.40 a
5	22.05 c	1.05 c	8.65 b	1.00 a	16.70 bc	70.63 a	13.27 a	24.41 b	25.99 b
10	21.79 c	1.09 c	8.95 b	1.10 a	15.65 c	69.74 a	13.16 a	24.72 b	22.62 b
15	23.73 bc	5.67 a	12.60 ab	1.15 a	21.00 ab	55.85 a	14.39 a	29.85 ab	32.53 b
20	26.52 ab	1.72 b	12.05 ab	1.20 a	17.60 bc	51.10 a	15.46 a	24.50 b	36.23 b
Troyer citrange									
0	27.03 a	2.86 ab	13.75 a	2.15 a	13.25 b	38.41 b	15.46 a	41.63 a	47.43 ab
5	27.67 a	2.75 b	13.35 a	2.10 a	14.35 b	35.02 b	10.15 ab	37.07 a	36.56 b
10	30.98 a	2.75 b	15.85 a	2.35 a	16.35 a	42.30 b	10.93 ab	36.95 a	62.35 a
15	31.57 a	3.05 ab	14.50 a	2.25 a	13.95 b	44.25 b	6.91 b	36.60 a	40.47 ab
20	31.86 a	3.44 a	17.85 a	2.30 a	13.40 b	77.73 a	13.76 ab	39.15 a	60.30 ab
Volkamer lemon									
0	23.79 c	1.98 c	17.40 bc	1.90 ab	16.75 a	45.40 c	15.14 a	36.97 c	47.00 c
5	30.99 bc	2.55 b	15.55 c	1.90 ab	18.55 a	44.68 c	15.64 a	44.13 b	46.35 c
10	37.56 ab	3.23 a	19.65 ab	1.80 ab	17.45 a	80.76 bc	17.96 a	55.95 a	79.09 a
15	38.28 ab	3.58 a	22.50 a	2.05 a	15.85 a	104.52 ab	16.22 a	48.09 b	56.47 bc
20	41.57 a	3.66 a	19.75 ab	1.60 b	16.15 a	123.70 a	17.91 a	37.49 c	70.52 ab

For each rootstock and each parameter, means with the same letter were not significantly different at *p* < 0.05, using the Duncan’s test. * Fe data were obtained by Pestana et al. (2011) [19].

**Table 3:** Macronutrient (mg g^−1^ DW) and micronutrient (µg g^−1^ DW) concentrations at the end of the experiment in roots of five citrus rootstocks grown in five Fe concentrations: 0, 5, 10, 15, and 20 µM.

Treatments [µM Fe]	N	P	Ca	Mg	K	Fe	Cu	Zn	Mn
Sour orange									
0	58.0 a	2.4 c	12.3 b	1.15 b	5.1 b	63.2 bc	116.3 a	97.0 a	110.1 c
5	67.2 a	1.7 c	7.2 b	1.15 b	5.0 b	53.6 c	67.6 bc	71.6 a	94.7 c
10	49.7 a	3.4 bc	11.0 b	1.50 bc	5.9 b	78.8 bc	80.5 b	94.4 a	100.1 c
15	42.7 a	14.0 a	30.1 a	2.40 a	6.9 ab	84.8 ab	60.5 bc	97.2 a	432.6 b
20	54.7 a	7.9 b	14.0 b	1.95 ab	8.5 a	110.6 a	46.2 c	82.8 a	526.7 a
Carrizo citrange									
0	29.82 b	3.90 b	3.85 b	1.20 b	5.65 b	48.91 b	132.53 a	129.56 b	156.79 c
5	31.06 b	3.79 b	3.75 b	1.45 ab	6.00 b	83.63 ab	104.43 ab	208.12 b	246.60 c
10	33.53 ab	8.48 a	3.55 b	1.65 a	12.30 a	108.75 ab	129.83 a	315.25 a	373.80 b
15	48.42 a	4.82 b	14.75 a	1.65 a	8.00 b	99.30 ab	76.10 b	190.34 b	372.55 b
20	30.69 b	4.80 b	3.75 b	1.60 a	8.80 b	146.84 a	69.02 b	174.32 b	693.88 a
*C. macrophylla*									
0	43.62 a	1.35 c	6.05 bc	1.55 a	10.80 a	72.95 c	288.03 a	129.15 a	867.85 a
5	32.84 a	1.50 bc	9.10 ab	1.35 a	8.10 a	193.92 a	137.79 b	87.00 ab	286.49 b
10	44.16 a	2.59 bc	5.75 c	1.25 a	8.30 a	167.49 a	94.31 b	83.03 b	130.42 b
15	35.85 a	3.62 b	10.75 a	1.40 a	8.80 a	119.95 b	79.17 b	63.13 b	128.34 b
20	32.26 a	7.59 a	11.10 a	1.40 a	8.00 a	135.54 b	76.50 b	65.48 b	228.83 b
Troyer citrange									
0	25.22 a	2.77 a	4.55 b	1.20 b	6.40 b	39.16 c	217.77 a	141.32 a	269.92 ab
5	27.61 a	2.07 b	5.35 ab	1.30 ab	7.30 b	92.15 ab	78.26 b	92.59 b	115.97 b
10	25.97 a	2.92 a	6.20 a	1.65 a	8.35 b	123.44 a	50.66 cd	79.00 b	316.02 a
15	28.27 a	2.51 ab	6.25 a	1.50 ab	10.35 a	87.08 b	34.99 d	97.76 b	141.85 b
20	24.30 a	3.10 a	5.40 ab	1.45 ab	11.45 a	108.13 ab	60.16 bc	105.35 b	389.78 a
Volkamer lemon									
0	31.27 b	2.68 c	8.30 a	1.50 b	7.65 b	71.52 a	174.89 a	61.00 c	116.36 b
5	41.32 ab	3.10 c	3.70 b	1.25 b	7.70 b	92.47 a	113.67 b	149.02 b	237.58 b
10	45.82 a	5.20 a	5.25 b	2.25 a	20.90 a	111.54 a	80.58 c	307.12 a	825.71 a
15	50.23 a	4.47 ab	8.05 a	2.35 a	24.20 a	95.97 a	73.04 c	188.08 b	351.90 b
20	48.55 a	4.23 b	6.90 a	1.65 b	19.90 a	128.50 a	60.47 c	79.82 c	788.18 a

For each rootstock, Fe concentration in the nutrient solution, and each parameter, means of at least three replicates with the same letter were not significantly different at *p* < 0.05, using the Duncan’s test.

## Data Availability

The data that support the findings of this study are available from the corresponding author upon reasonable request.

## Supplementary Materials

The following supporting information can be downloaded at: https://www.mdpi.com/article/10.3390/plants12183252/s1, Figure S1: Aspect of citrus rootstocks plants for each treatment at the end of the experiment; Table S1: Shoot and root dry weight (DW in g), root-to-shoot ratio (in DW basis), and total DW of five citrus rootstocks at the beginning of the experiment; Table S2: Loadings of the PCA for nutrient composition and leaf chlorosis parameters for five citrus rootstocks; Table S3: Pearson’s correlations between PCA variables: leaf chlorosis parameters and nutrients in shoots (S) and in roots (R) of five citrus rootstocks; Table S4: Macronutrient (mg) and micronutrient (µg) contents at the end of the experiment in five citrus rootstocks grown with five Fe concentrations in nutrient solution: 0, 5, 10, 15, and 20 µM; Table S5: Loadings of the PCA for total nutrient content and leaf parameters for five Fe levels in nutrient solution considering rootstocks as one. DW—shoot plus root dry weights.
