# Correction: Pestana et al. Nutritional Performance of Five Citrus Rootstocks Under Different Fe Levels. *Plants* 2023, *12*, 3252

**DOI:** 10.3390/plants15040593

**Published:** 2026-02-13

**Authors:** Maribela Pestana, Pedro García-Caparrós, Teresa Saavedra, Florinda Gama, Javier Abadía, Amarilis de Varennes, Pedro José Correia

**Affiliations:** 1 MED—Mediterranean Institute for Agriculture, Environment and Development, CHANGE–Global Change and Sustainability Institute, Faculty of Science and Technology, University of Algarve, Campus of Gambelas, Building 8, 8005-139 Faro, Portugal; tmsaavedra@ualg.pt (T.S.); pcorreia@ualg.pt (P.J.C.); 2 Department of Agronomy, Higher Engineering School, University of Almeria, Agrifood Campus of International Excellence CeiA3, Ctra. Sacramentos/n, La Cañada de San Urbano, 04120 Almería, Spain; 3 GreenCoLab—Associação Oceano Verde, University of Algarve, Campus de Gambelas, 8005-139 Faro, Portugal; 4 Plant Biology Department, Estación Experimental de Aula Dei, CSIC, Av. Montañana 1005, 50059 Zaragoza, Spain; 5 Instituto Superior de Agronomia, University of Lisbon, Tapada da Ajuda, 1349-017 Lisbon, Portugal; amdevarennes@gmail.com

In the original publication [1], the funding by **National Funds through FCT-Foundation for Science and Technology**, **UIDB/05183/2020 (https://doi.org/10.54499/UIDB/05183/2020)** and **LA/P/0121/2020 (https://doi.org/10.54499/LA/P/0121/2020)** provided to ** Maribela Pestana** was not included. The authors state that the scientific conclusions are unaffected. This correction was approved by the Academic Editor. The original publication has also been updated.
