# Supplementary material for: Nutritional Performance of Five Citrus Rootstocks under Different Fe Levels
Source: Plants (Basel). 2023 Sep 13;12(18):3252. doi: 10.3390/plants12183252 (PMC10535202; doi:10.3390/plants12183252)
Supplement: Supplementary file 1 [file plants-12-03252-s001.zip › plants-2554728-supplementary.pdf]

Supplementary Information

Table S1: Shoot and root dry weight (DW in g), root to shoot ratio (in DW basis), and total DW of five citrus rootstocks at the beginning of the experiment.

Rootstock	Initial Leaf CHL (µmol m^{-2})	Shoot DW (g)	Root DW (g)	Root/Shoot	Total DW (g)
**Sour orange**	349.9 ± 78.0	0.66 ± 0.07	0.20 ± 0.02	0.31 ± 0.14	0.87 ± 0.04
**Carrizo citrange**	455.9 ± 73.5	0.40 ± 0.02	0.22 ± 0.01	0.55 ± 0.10	0.62 ± 0.02
C. macrophylla	399.9 ± 63.1	0.30 ± 0.02	0.09 ± 0.01	0.30 ± 0.07	0.39 ± 0.01
**Troyer citrange**	460.2 ± 93.1	0.29 ± 0.02	0.18 ± 0.01	0.62 ± 0.13	0.47 ± 0.02
**Volkamer lemon**	387.1 ± 70.1	0.36 ± 0.02	0.12 ± 0.01	0.35 ± 0.08	0.48 ± 0.02

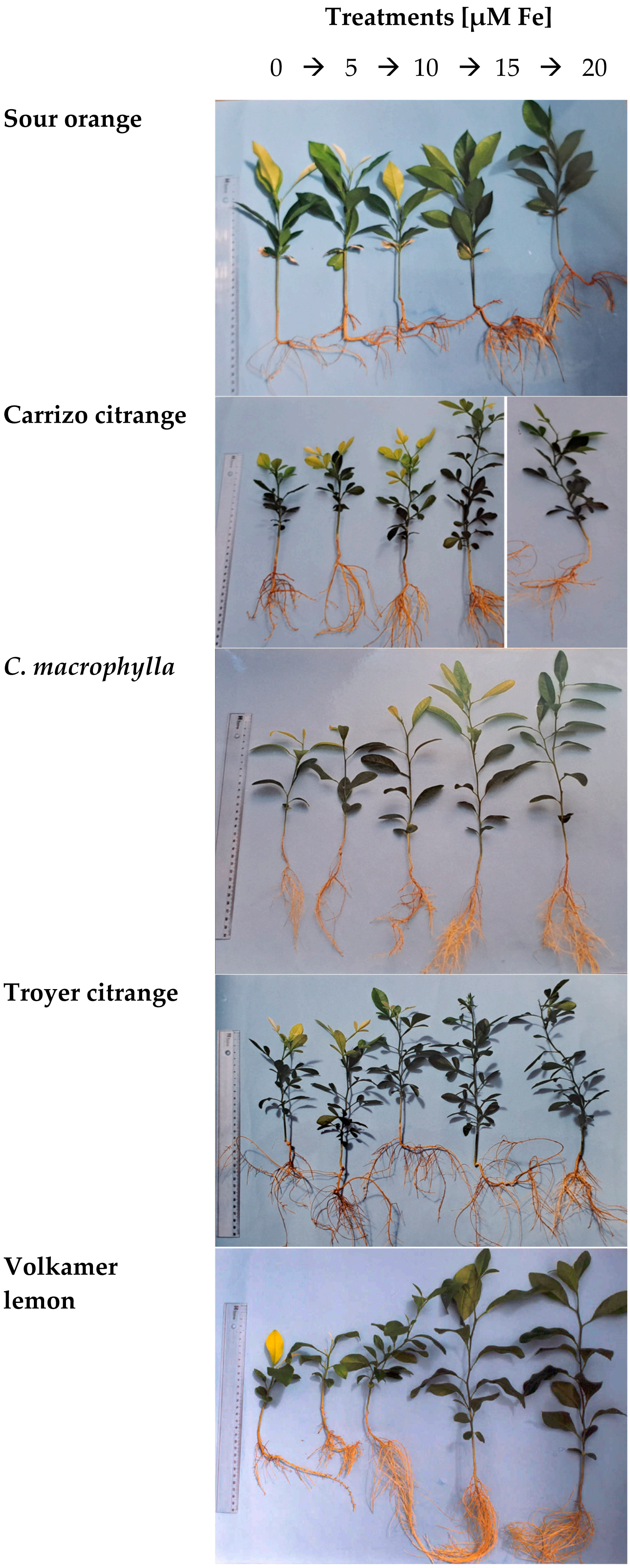

Figure S1: Aspect of citrus rootstocks plants for each treatment at the end of the experiment.

Table S2: Loadings of the PCA for nutrient composition and leaf chlorosis parameters for five citrus rootstocks. Significant values are in bold.

Variables	PC1	PC2
**CHL**	0.344	**0.635**
**FCR**	0.373	0.016
**Leaf area**	-0.179	**0.888**
**S-DW**	-0.309	**0.814**
**N-S**	**0.834**	0.276
**P-S**	**0.758**	0.037
**Ca-S**	**0.839**	0.083
**Mg-S**	0.562	0.010
**K-S**	0.498	-0.355
**Fe-S**	0.498	0.483
**Cu-S**	**0.775**	-0.400
**Zn-S**	**0.834**	-0.266
**Mn-S**	**0.885**	-0.261
**N-R**	-0.155	0.358
**P-R**	0.215	0.535
**Ca-R**	-0.282	0.519
**Mg-R**	0.476	**0.636**
**K-R**	0.620	0.410
**Fe-R**	0.070	0.141
**Cu-R**	0.085	**-0.778**
**Zn-R**	**0.757**	-0.090
**Mn-R**	**0.678**	0.212
**R-DW**	0.007	0.606

Table S3: Pearson's correlations between PCA variables: leaf chlorosis parameters and nutrients in shoots (S) and in roots (R) of five citrus rootstocks.

	CHL	FCR	N-S	P-S	Ca-S	Mg-S	K-S	Fe-S	Cu-S	Zn-S	Mn-S	N-R	P-R	Ca-R	Mg-R	K-R	Fe-R	Cu-R	Zn-R	Mn-R	R-DW	S-DW	Leaf area
**CHL**	1																						
**FCR**	0.196	1																					
**N-S**	0.399*	0.183	1																				
**P-S**	0.326	0.242	**0.700****	1																			
**Ca-S**	0.288	0.292	**0.763****	**0.536****	1																		
**Mg-S**	0.092	-0.011	**0.654****	**0.609****	**0.587****	1																	
**K-S**	-0.058	**0.627****	0.112	0.277	0.376	-0.105	1																
**Fe-S**	**0.562****	-0.014	**0.473***	0.217	**0.466***	-0.033	0.161	1															
**Cu-S**	0.123	0.192	**0.484***	**0.543****	**0.560****	0.252	**0.633****	0.366	1														
**Zn-S**	0.036	0.261	**0.704****	**0.706****	**0.684****	**0.684****	0.309	0.108	**0.614****	1													
**Mn-S**	0.083	**0.405***	**0.720****	**0.656****	**0.761****	**0.532****	**0.530****	0.228	**0.698****	**0.840****	1												
**N-R**	0.088	-0.012	0.049	-0.257	-0.059	**-0.454***	-0.178	0.177	-0.248	-0.272	-0.162	1											
**P-R**	0.288	0.018	0.292	0.374	0.024	0.083	0.021	0.272	-0.014	0.033	0.020	0.094	1										
**Ca-R**	0.052	0.031	-0.102	-0.209	-0.220	-0.282	-0.130	0.033	**-0.441***	-0.381	-0.295	0.358	0.677**	1									
**Mg-R**	0.382	0.173	**0.452***	0.199	**0.434***	0.150	0.132	**0.552****	0.085	0.210	0.231	0.250	**0.637****	**0.456***	1								
**K-R**	**0.511****	0.236	**0.482***	0.245	**0.590****	0.119	0.231	**0.756****	**0.451***	0.277	0.315	0.163	0.103	-0.165	**0.653****	1							
**Fe-R**	0.284	0.023	-0.056	0.033	-0.199	-0.318	0.244	0.443*	0.170	-0.127	-0.051	-0.126	0.042	-0.022	0.139	0.238	1						
**Cu-R**	**-0.528***	0.066	-0.244	-0.129	0.046	-0.139	**0.415***	-0.263	0.410*	0.159	0.307	-0.141	-0.288	-0.246	-0.287	-0.153	-0.318	1					
**Zn-R**	0.186	0.138	**0.657****	**0.618****	**0.455***	**0.493***	0.119	0.169	**0.569****	**0.771****	**0.632****	-0.020	0.230	-0.288	0.394	**0.428***	-0.047	0.106	1				
**Mn-R**	0.323	0.388	**0.489***	0.311	**0.551****	0.014	**0.512****	,604**	**0.475***	0.329	**0.619****	0.125	0.264	-0.004	**0.557****	**0.583****	0.236	0.136	0.386	1			
**R-DW**	0.225	-0.023	0.286	0.177	0.160	**0.555****	**-0.419***	-0.021	-0.393	0.032	-0.099	-0.150	0.290	0.177	0.240	0.037	-0.350	**-0.534****	-0.070	-0.049	1		
**S-DW**	**0.423***	-0.017	-0.023	-0.189	-0.081	-0.089	**-0.479***	0.113	**-0.599****	**-0.437***	**-0.465***	0.356	0.187	0.345	0.260	0.198	-0.154	**-0.600****	-0.338	-0.042	**0.658****	1	
**Leaf area**	**0.505***	0.102	0.075	-0.091	-0.083	-0.078	-0.324	0.302	**-0.492***	**-0.412***	-0.346	0.239	0.284	0.377	0.394	0.261	0.199	**-0.739****	-0.309	0.119	**0.615****	**0.817****	1

* Significant difference at $P < 0.05$. **. Significant difference at $P < 0.01$.

Table S4: Macronutrients (mg) and micronutrients (μg) contents at the end of the experiment in five citrus rootstocks grown with five Fe concentrations in nutrient solution: 0, 5, 10, 15 and 20 μM.

**Treatments [μM Fe]**	**N**	**P**	**Ca**	**Mg**	**K**	**Fe**	**Cu**	**Zn**	**Mn**
**Sour orange**									
0	49.5	2.7	18.3	5.7	12.7	67.7	45.7	57.9	74.1
5	66.8	2.7	19.3	5.4	19.5	69.1	46.0	64.4	70.7
10	68.9	4.0	22.8	7.3	20.1	98.0	48.5	81.4	71.8
15	67.5	11.2	35.6	20.3	23.8	136.9	46.9	92.9	296.3
20	66.6	7.4	23.4	9.4	22.0	162.6	33.1	73.8	329.7
**Carrizo citrange**									
0	30.4	3.4	10.9	2.6	10.5	54.4	60.0	73.2	95.9
5	40.4	5.0	14.0	3.4	13.9	72.3	57.3	153.0	176.7
10	40.8	7.8	11.0	3.0	15.2	80.7	65.2	161.7	207.1
15	54.9	6.4	22.0	8.3	17.0	81.2	46.3	133.1	250.5
20	47.7	7.3	18.8	3.4	18.7	128.9	43.6	117.5	367.9
C. macrophylla									
0	28.7	1.7	13.4	2.2	18.8	49.3	80.8	60.1	281.0
5	18.8	1.1	6.7	2.1	11.4	75.3	31.4	29.4	64.3
10	26.7	1.1	7.8	2.3	13.1	92.4	33.7	38.9	49.7
15	43.6	10.0	19.8	5.8	29.2	117.3	50.0	62.3	145.2
20	36.2	2.9	15.2	4.9	19.4	94.6	40.7	45.5	112.2
**Troyer citrange**									
0	39.0	4.2	15.2	4.5	15.8	57.3	136.2	117.4	194.8
5	43.1	3.9	16.3	5.1	18.4	86.6	54.0	88.9	101.5
10	50.1	4.8	21.5	6.3	23.3	119.4	41.7	87.6	253.7
15	63.3	6.0	24.8	7.2	26.6	118.6	31.6	114.3	147.6
20	57.3	6.4	26.9	6.2	24.6	167.2	53.8	114.2	310.2
**Volkamer lemon**									
0	27.8	2.3	15.8	4.0	15.1	56.7	65.7	47.0	71.8
5	44.6	3.6	15.7	3.2	20.1	77.2	58.7	98.7	135.3
10	55.4	5.7	21.7	3.8	25.6	124.3	49.4	175.7	401.1
15	71.7	6.6	32.3	6.3	31.3	176.9	54.2	147.6	233.5
20	83.7	6.7	31.7	5.7	33.0	241.1	55.8	93.5	494.9

Table S5: Loadings of the PCA for total nutrient content and leaf parameters for five Fe levels in nutrient solution considering rootstocks as one. DW – total dry weight (shoot plus root). Significant values are in bold.

Variables	PC1	PC2
**CHL**	**0.971**	0.240
**FCR**	-0.053	**0.999**
**Leaf area**	**0.971**	0.240
**DW**	**0.929**	0.370
**N**	**0.929**	0.370
**P**	**0.929**	0.370
**Ca**	0.552	**0.833**
**Mg**	**0.929**	0.370
**K**	**0.929**	0.370
**Fe**	**0.971**	0.240
**Cu**	**-0.941**	-0.339
**Zn**	**0.979**	-0.204
**Mn**	0.570	**0.821**
